# Cortical neurodynamics changes mediate the efficacy of a personalized neuromodulation against multiple sclerosis fatigue

**DOI:** 10.1038/s41598-019-54595-z

**Published:** 2019-12-03

**Authors:** Camillo Porcaro, Carlo Cottone, Andrea Cancelli, Paolo M. Rossini, Giancarlo Zito, Franca Tecchio

**Affiliations:** 1 LET’S – ISTC – CNR, Rome, Italy; 2 S. Anna Institute and Research in Advanced Neurorehabilitation (RAN), Crotone, Italy; 3 Department of Information Engineering Università Politecnica delle Marche, Ancona, Italy; 4 Movement Control and Neuroplasticity Research Group, Department of Kinesiology, KU Leuven, Leuven, 3001 Belgium; 5 Area di Neuroscienze, IRCCS San Raffaele Pisana, Rome, Italy; 6 Innovation and Pharmaceutical Strategy Division, Italian Medicines Agency (AIFA), Rome, Italy

**Keywords:** Neuroscience, Multiple sclerosis, Mathematics and computing

## Abstract

The people with multiple sclerosis (MS) often report that fatigue restricts their life. Nowadays, pharmacological treatments are poorly effective accompanied by relevant side effects. A 5-day transcranial direct current stimulation (tDCS) targeting the somatosensory representation of the whole body (S1) delivered through an electrode personalized based on the brain MRI was efficacious against MS fatigue (FaReMuS treatment). This proof of principle study tested whether possible changes of the functional organization of the primary sensorimotor network induced by FaReMuS partly explained the effected fatigue amelioration. We measured the brain activity at rest through electroencephalography equipped with a Functional Source Separation algorithm and we assessed the neurodynamics state of the primary somatosensory (S1) and motor (M1) cortices via the Fractal Dimension and their functional connectivity via the Mutual Information. The dynamics of the neuronal electric activity, more distorted in S1 than M1 before treatment, as well as the network connectivity, altered maximally between left and right M1 homologs, reverted to normal after FaReMuS. The intervention-related changes explained 48% of variance of fatigue reduction in the regression model. A personalized neuromodulation tuned in on specific anatomo-functional features of the impaired regions can be effective against fatigue.

## Introduction

Fatigue occurs in up to 80% of people with multiple sclerosis (MS), and approximately two-thirds of people with MS report unusually prolonged daily tiredness as their most troubling symptom^1^. Fatigue can be managed with only minimal relief to date, and all treatments are often burdened with relevant adverse events^2^. Behind the fatigue in the MS there is not a single etiology, which instead involves functional and structural impairments of the primary sensorimotor networks^3^.

In two independent groups of people with MS^4,5^, we successfully tested the efficacy of a transcranial direct current stimulation (tDCS) intervention against MS fatigue, while bilaterally targeting the whole-body somatosensory areas (S1). To do this, we developed a personalized tDCS electrode, shaped on the individual MRI-derived outline of the central sulcus and properly positioned to selectively target the whole-body S1 representation^6^. A higher efficacy of this approach with respect to the use of a non-personalized electrode had already been experimentally demonstrated for the whole-body S1^7^, and further confirmed by recent modeling studies^8,9^. The treatment protocol (called FaReMuS - Fatigue Relief in Multiple Sclerosis) implements a 5-day anodal tDCS through a personalized S1 electrode against an occipital cathode. In support of the appropriateness of the selected target in determining the treatment efficacy against MS fatigue, the negative outcome of two other tDCS trials can be considered, in which every parameter were identical less except for the electrode shape and position^10,11^. Among the 18 patients enrolled for the present investigation, the overall amelioration after real stimulation (Table 1) was of 30% of the baseline level, with a Cohen’s d coefficient^12^ equal to 1.1 (difference between the two pairwise means of mFIS before and after treatment divided by the pooled standard deviations). Reference values indicate that a coefficient of 0.2 indicates a small effect size (ES), 0.5 a medium ES and higher than 0.8 large ESs, thus clearly evidencing a large effect, consistent with the further classification by Sawilowsky^13^, who indicated as very large effects those corresponding to Cohen’s d above 1.2. We need to underline that, despite personalizing the intervention, we observed a relevant variability in the treatment efficacy, with 12 out of 18 people being Responder, defined as ameliorating the fatigue level more than 20% of baseline. Although our sample size does not have enough power to test the main effect Duration, we can say that the mean duration of the effect was more than 2 months for the Responders.

**Table 1:** MS patient demographic and clinical profile.

								mFIS					
Sex		Age	DD	ARR	EDSS	BDI	Lesion load	Pre Real	Post Real	% Real	Pre Sham	Post Sham	% Sham
14F, 4M	Ave/Med	44.5	6.9	0	*1.1*	10.4	0.003	45.6	32.5	29.9	44.9	41.4	7.5
	SD/ Ran	10.4	5.5	[0–2]	[0–3.5]	3.1	0.002	[31,66]	[9,55]	[2,76]	[30,67]	[26,69]	[−11,37]

F = female, M = male; mean (Ave) or median (Med, in italics) and SD = standard deviations or ranges (Ran) [min, max] across the 18 MS patients’ group of: DD = disease duration; ARR = annual relapse rate; Scores of: EDSS = Expanded Disability Status Scale, BDI = Beck Depression Inventory; Lesion load: volume of the lesion divided by the intra-cranial volume, as assessed by NeuroQuant algoritms (CorTechs Labs, Inc. San Diego, CA, USA); mFIS = modified fatigue impact scale; Pre and Post FaReMuS and the percentage change with respect to baseline (%, defined as (pre-post)/pre) for Real and Sham stimulations.

**Figure 1:**
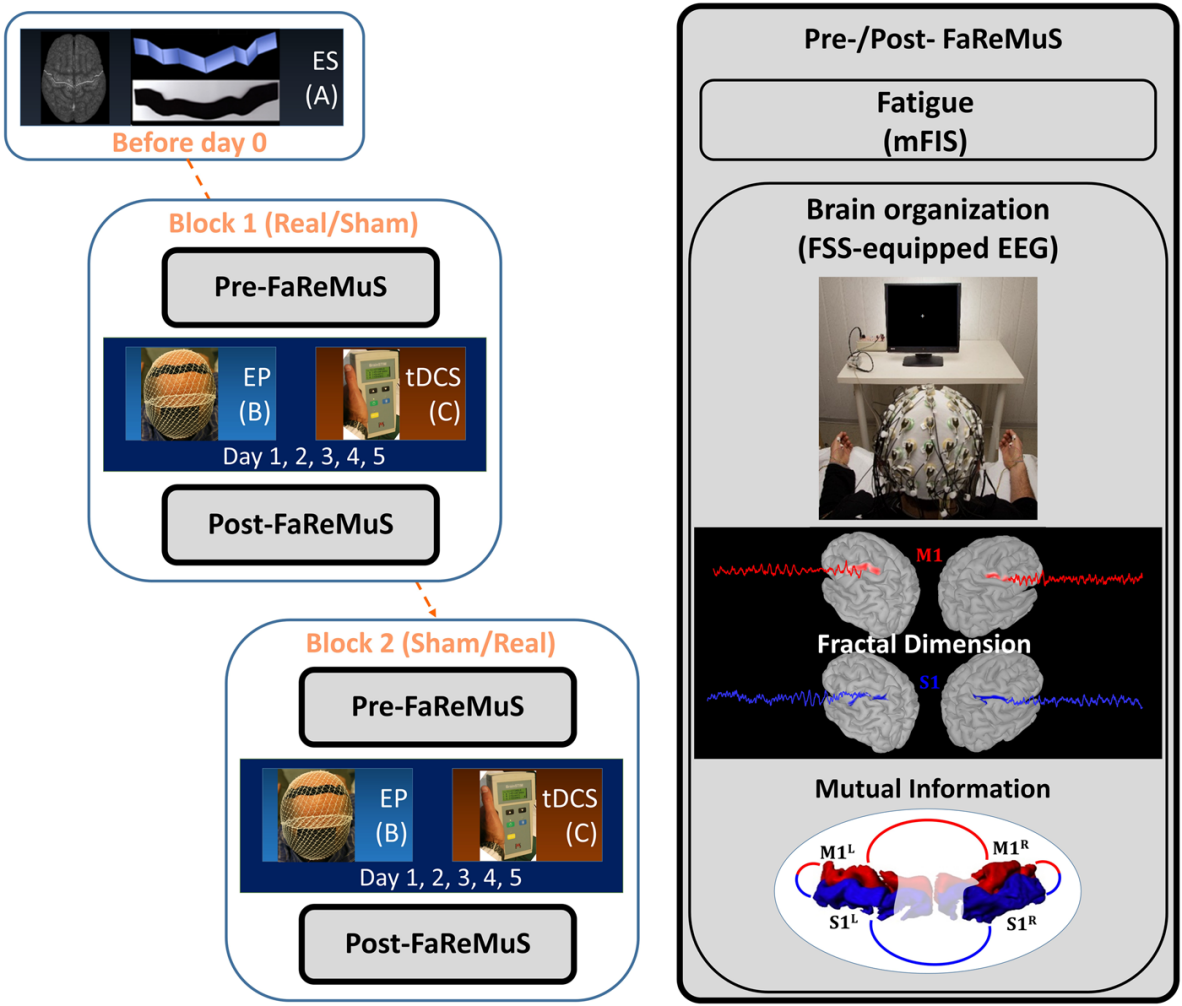
Randomized clinical trial design and FaReMuS effect testing.  Experimental procedure in each person with MS undergoing FaReMuS. Left: Individual brain MRI-based personalized electrode shaping (ES, A) performed once for each person with MS. In blocks 1 and 2 (equal, but each stimulation is either Real or Sham), after the data collection (Pre-FaReMuS), the electrode positioning (EP, B) is executed for each tDCS stimulation (C) repeated for 5 days of treatment. In the day of the last tDCS, we performed the data collection (Post-FaReMuS). Right: The data collection (Pre- and Post-FaReMuS) includes the fatigue level assessment via mFIS and the EEG for the brain effects. We represented the measures used for the resting state local activity (fractal dimension of the primary sensory and motor cortices) and the resting state functional connectivity between them (mutual information).

By the present proof of concept study, we tested the working hypothesis that FaReMuS modifies the functional organization of the brain networks involved in MS-related fatigue contributing to its efficacy. In this way, we aim at deepen the understanding of the phenomena subtending fatigue and its relief. We deployed a two-step approach on the electroencephalographic (EEG) signal data, collected before and after Real and Sham stimulations (Fig. 1), answering these questions: 1. Does FaReMuS modulate the dynamics of the neuronal electrical activity, called neurodynamics, of sensorimotor networks? In particular, does it act more on S1’s or on M1’s activity? 2. Is there a correlation between such changes and the extent of amelioration of fatigue symptoms?.

**Figure 2:**
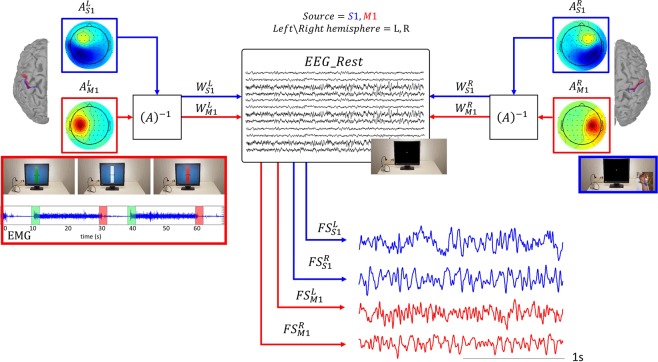
S1 and M1 resting state activity as derived by FSS-equipped EEG. We detail here the experimental settings required for FSS application. Motor condition for FSS-identify M1 source (red code): The experiment was executed with the left and right hand separately. People performed a handgrip against resistance of a semi-compliant air-bulb, connected to a digital board hosting in-house-developed electronics that recorded the exerted pressure giving to the subject a visual feedback (Interactive Pressure Sensor, InPresS). After determining right and left handgrip maximum voluntary contraction (MVC), we waited at least 2 minutes with the subject at rest to avoid fatigue effects. A Dell PC, using InPresS, controlled the stimulus presentation. We asked the subjects to maintain the isometric contraction from the go- to the stop-visual signal (green and red image on the screen, inset on the left). Contraction periods lasted 20 seconds, intermingled by 10 s periods of rest to avoid task-induced fatigue (see the EMG from the opponens pollicis muscle in the inset). A vertical, white force gauge was centrally displayed upon a light blue background throughout the whole experiment. Two black segments aside the gauge indicated the required force level (5% of MVC). A horizontal black force indicator bar appeared centrally in the force gauge upon trial onset. The vertical position of this horizontal indicator provided continuous visual feedback to the subject about the exerted contraction force. At the trial offset (stop-signal red image), subjects terminated the contraction and completely relaxed their hand till the start of the successive contraction. Sensory condition for FSS-identify S1 source (blue code): Separately for left and right hands, we collected the cortical responses to electrical stimulation (constant current isolated stimulator DS3, Digitimer Ltd, Hertfordshire, England) delivered at repetition rate of 4.4 Hz to the median nerve at the wrist of either the left or right hand (square wave pulses, 0.2 ms duration, cathode proximal). The stimulus intensity was set just above the motor threshold, inducing a painless thumb twitch, which the experimenter visually monitored. We collected one thousand sweeps for each side (5 min altogether).

The specific activities of S1 and M1 were disentangled by the Functional Source Separation (FSS) algorithm^14,15^. In particular, the FSS version herein used identifies the neuronal pools of the hand representations within primary cortical S1 and M1^16^, as we already demonstrated^17–23^. Because fatigue is a chronic condition, and its long-lasting amelioration by FaReMuS is achieved independently from specific tasks, we were mainly interested in quantifying the brain effects of FaReMuS in resting state. To this aim, we used the fractal dimension (FD)^24^ of FSS-identified FS_S1_ and FS_M1_ neuronal activities in resting state with open eyes before and after treatment (Fig. 2). FD already proved to be a good candidate for assessing the brain networks state and functionality^25–28^ and for typifying cortical districts^22,29^. To quantify the resting state functional connectivity among the nodes, we calculated the mutual information (MutInf)^30^ in assessing the S1-M1 communication in each hemisphere, as well as the functional communication between hemispheric homologs^31,32^. We selected FD for the neuronal pools’ state and, among the multiple relevant approaches^33^, MutInf for their connectivity, believing that complex-system measures are proper to describe the physiology of neuronal electric activity, whose dynamics display hugely complex temporal structures^34,35^.

## Material and Methods

### Study design

To assess the effects of FaReMuS on the primary sensorimotor network organization, we investigated the EEG-derived and FSS-identified activities of FS_S1_ and FS_M1_ hand representation at rest, before and after FaReMuS. FaReMuS treatment was tested in a clinical setting with a randomized, double-blind, Sham-controlled, crossover design (Fig. 1). The effects on fatigue, as measured on patients by the Modified Fatigue Impact Scale (mFIS), were already described in two independent trials^4,5^. To assess the individual skills in the fine hand motor control we collected the 9-Hole Peg test executed with each hand before and after FaReMuS. According to a pre-defined time schedule, at T0 (pre-FaReMuS, the day of first tDCS application and before the stimulation) and at T1 (post-FaReMuS treatment, 5–7 days after t0, see Fig. 1), each patient, in addition to scoring of the mFIS, underwent the collection of the EEG and EMG (see Figs. 1 and 2).

People were recruited whether diagnosed with relapsing MS according to McDonalds Criteria^36^ and complying with the eligibility criteria as follows.

#### Inclusion criteria

- absence of clinical or radiological evidence of disease activity (NEDA) for at least 3 months preceding the study;
- low degree of disability as estimated by Extended Disability Scale Score (EDSS, Kurtzke 1983) < 3,5;
- fatigue as estimated by mFIS > 30.

#### Exclusion criteria were

- Current or prior (within less than 12 weeks before enrolment) exposure to psychotropic drug(s) (antidepressant, anxiolytic, antipsychotics, anticonvulsants, myorelaxant drugs);
- Coexistence of other condition(s) potentially associated with fatigue (i.e. anemia, pregnancy);
- Current or prior (within less than 4 weeks before enrolment) exposure to anti-fatigue products;
- History of epilepsy.

All methods were carried out in accordance with relevant guidelines and regulations. All experimental protocols were approved by Ethics Committee of the ‘S. Giovanni Calibita’ Fatebenefratelli Hospital in Rome (n. 22/2012). All patients signed the informed consent form before their enrollment.

### Regional personalized electrode (RePE) shaping and tDCS treatment (5-day treatment)

As detailed in^4,5^ individualized electrode was shaped derived from brain MRI in each patient, positioned and tDCS applied for 15 minutes every day for 5 days.

### Electrophysiological data recordings

EEG signals were recorded using a 64-channel actiCHamp System (Brain Products GmbH, Munich, Germany) with electrodes positioned in the sites of 10–10 EEG International System. Electrode impedances were maintained below 5 kΩ. Surface electromyogram (EMG) of the right and left opponens pollicis muscles was recorded with a belly-tendon montage (2.5 cm inter-electrode distance) by Ag-AgCl cup electrodes. With the same kind of electrodes we collected electrooculogram (EOG) and electrocardiogram (ECG) to control eye blinking and cardiac interferences. We sampled EEG, EOG, ECG and EMG at 5 kHz (pre-sampling analogical bandpass filtering 0.1–2000 Hz) and collected them for off-line processing.

### Experimental procedures for EEG/EMG data collection, application of FSS and identification of left and right S1 and M1 sources

As detailed in^22^ we applied the FSS algorithm to EEG and EMG (Fig. 2) to enable the study of the S1 and M1 activities in resting state.

### Primary sensorimotor network nodes’ ongoing activity

We calculated the fractal dimension (FD) for each source resting state activity using the algorithm proposed by Tomoyuki Higuchi to calculate fractal dimension directly in the time domain, without transforming the time series to the frequency domain and without embedding data in a phase space^24^.

### Functional connectivity among primary sensorimotor network nodes

We calculated the Mutual Information (MutInf,^30^) to assess resting state functional connectivity between the FSs’ activities as:

$$MI(X;Y)=\sum _{y\in Y}\sum _{x\in X}p(x,y)log(\frac{p(x,y)}{p(x)p(y)})$$

where the two discrete random variables X and Y are FSs(t), *p(x,y)* is the joint probability distribution function of X and Y, and *p(x)* and *p(y)* are the marginal probability distribution functions of X and Y respectively. MutInf is equal to zero if and only if X and Y are independent random variables. We have used the Freedman-Diaconis rule to estimate the number of bins used for discretization^37^. Specifically, we considered the MutInf in each hemisphere between $F{S}_{S1}$ and $F{S}_{M1}$ and between hemispheric homologs ($F{S}_{M1}^{L}$ and $F{S}_{M1}^{R}$ and similarly for $F{S}_{S1}$).

### Statistical analysis

We checked whether variables’ distribution fitted the Gaussian according to the Shapiro–Wilk statistic. We reported a result for the effect significance p < 0.050 and suggestions for p < 0.100.

We designed ad hoc statistical models to answer whether the improvement of fatigue in MS patients was accompanied by changes in the sensorimotor cortex physiology after FaReMuS.

1. To investigate whether FaReMuS changed the brain organization, we tested the FaReMuS-related changes of local activity and functional connectivity of primary sensorimotor cortices. For the local activity, the Full Model Analysis of Variance (ANOVA) for repeated measures included *FaReMuS Treatment* (Pre, Post), *Cortical District* ($F{S}_{S1}$, $F{S}_{M1}$) and *Hemisphere* (Left, Right) as within-subject factors. We focused on FaReMuS Treatment*Cortical District effect to reveal different effects of the intervention upon FS_S1_ and FS_M1_. If so, we applied relative Reduced Models to investigate how the two cortical districts changed after FaReMuS.
  In the case of *FaReMuS* effect on the activities of sensorimotor network nodes, we compared the variable values before and after FaReMuS to those in healthy people (not submitted to neuromodulation).
  We executed the analysis first on Real FaReMuS data, and we checked what happened in Sham block of those aspects modified by Real stimulation.
  Furthermore, we checked whether the whole groups’ effects were confirmed in the two groups of 9 earliest and 9 latest recruited people.
2. A predictive model tested the extent of brain organization changes explaining part of the fatigue amelioration. The cortical parameters that showed a change following FaReMuS at step 1, underwent a preliminary correlative analysis with mFIS variations.

## Results

Eighteen subjects with MS suffering from fatigue entered the present study (Table 1) and were investigated before and after Real tDCS (18*2 = 36 EEG/EMG recordings). EEG recordings were not available for 5 subjects in Sham treatment, thus we executed the analysis in the cross-over design for 13 of the 18 subjects (13*4 conditions = 52 recordings).

### FaReMuS effects on neuronal dynamics of primary sensorimotor cortex

Shapiro-Wilk test indicated that none of the FDs among the four primary sensorimotor cortical sources ($F{S}_{S1}^{L}$, $F{S}_{S1}^{R},$ $F{S}_{M1}^{L}$, $F{S}_{M1}^{R}$) either before or after FaReMuS differed from a Gaussian distribution (p > 0.300 consistently).

**Figure 3:**
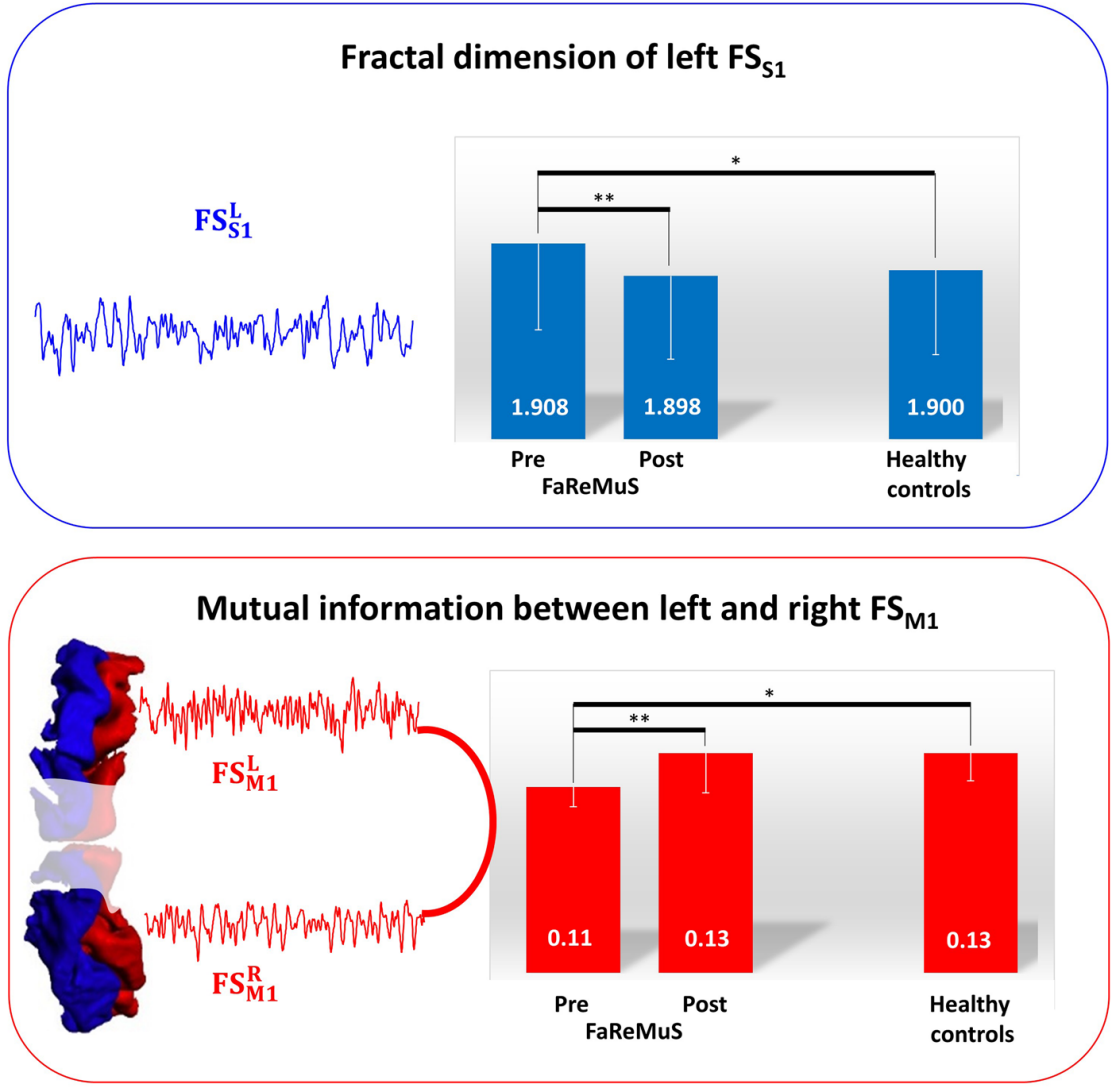
FaReMuS effects on the dynamic organization of the primary sensorimotor network. Representation of the factors mostly changed by FaReMuS. Top: the fractal dimension of the dynamics of the left FSS1 resting state activity. Bottom: the mutual information between left FSM1 and right FSM1 at rest. ^**^p<0.01; ^*^p <0.05.

We submitted these values to the planned ANOVA. A strong interaction effect *FaReMuS Treatment* * *Cortical District* [F(1,17) = 12.066, p = 0.003] indicated that the intervention had a distinct impact on FS_S1_ and FS_M1_. After repeating the analysis separately on the two cortical areas, a strong effect was found in *FaReMuS Treatment* * *Hemisphere* interaction [F(1,17) = 21.263, p < 0.001] for the S1 cortical district, indicating a diverse effect of FaReMuS in left and right FS_S1_. The corresponding ANOVAs in the each hemisphere evidenced a strong ***FaReMuS Treatment*** [F(1,17) = 11.064, p = **0.004**] effect for the **left hemispheric S1**, corresponding to a FD reduction of left FS_S1_ activity dynamics after FaReMuS treatment (Fig. 3). A weaker effect was also found in the right FS_S1_ (*FaReMuS Treatment* F(1,17) = 5.265, p = 0.035), corresponding to a FD increase of right FS_S1_ dynamics. The slight bilateral increase of FD in FS_M1_ only approached significance (*FaReMuS Treatment* F(1,17) = 4.201, p = 0.056, Table 2).

**Table 2:** Local dynamics of FS_S1_ and FS_M1_ before and after FaReMuS.

	Fractal dimension							
	Real				Sham			
	${{\bf{FS}}}_{{\bf{S}}{\bf{1}}}^{{\bf{L}}}$	${{\bf{FS}}}_{{\bf{S}}{\bf{1}}}^{{\bf{R}}}$	${{\bf{FS}}}_{{\bf{M}}{\bf{1}}}^{{\bf{L}}}$	${{\bf{FS}}}_{{\bf{M}}{\bf{1}}}^{{\bf{R}}}$	${{\bf{FS}}}_{{\bf{S}}{\bf{1}}}^{{\bf{L}}}$	${{\bf{FS}}}_{{\bf{S}}{\bf{1}}}^{{\bf{R}}}$	${{\bf{FS}}}_{{\bf{M}}{\bf{1}}}^{{\bf{L}}}$	${{\bf{FS}}}_{{\bf{M}}{\bf{1}}}^{{\bf{R}}}$
Pre FaReMuS	**1.908**	1.899	1.913	1.909	1.905	1.900	1.915	1.916
	*0.026*	*0.024*	*0.022*	*0.023*	*0.021*	*0.021*	*0.021*	*0.021*
Post FaReMuS	1.898*	1.908	1.916*	1.920	1.908	1.908	1.915	1.918
	*0.025*	*0.020*	*0.022*	*0.019*	*0.016*	*0.020*	*0.021*	*0.017*
Healthy controls	1.900*	1.898	1.919*	1.908				
	*0.025*	*0.024*	*0.024*	*0.021*				

Mean and *standard deviation* of fractal dimensions before and after treatment, for Real and Sham FaReMuS in fatigued MS patients. Healthy controls did not underwent stimulation. Asterisks refer to the comparison between S1 and M1 fractal dimensions: they differed in controls and post-FaReMuS while the differentiation was impoverished pre-FaReMuS. In bold, the values changed after with respect to before FaReMuS for p < 0.010.

When the same analysis was executed separately in the two groups of 9 patients each (earliest/latest recruited), the effects seen in the 18 patients sample showed significance for both groups (p < 0.100). No other effects emerged.

We tested the fractal dimension of the left FS_S1_ neurodynamics after Sham stimulation. The outcome of the within-subject factors ANOVA with *FaReMuS Treatment* (pre, post) and the *RealSham* (Real, Sham), suggested a different change of FD with respect to the stimulation delivered (FaReMuS Treatment*RealSham interaction effect [F(1,12) = 4.157, p = 0.064]), which as outlined above was clear after the Real stimulation (*FaReMuS Treatment* p = 0.004). On the other hand, such an effect was absent with Sham stimulation [F(1,12) = 0.162, p = 0.695]. Finally, no difference emerged when comparing the pre-treatment FD in the two Real/Sham blocks by paired sample t-test (p > 0.500 consistently).

With regard to the direction of changes among patients and healthy controls, the Fractal Dimensions before the treatment were included into a Full Model, together with *Cortical District* (FS_S1_, FS_M1_) and *Hemisphere* (Left, Right) as within-subject factors, and *HealthyFatiguedMS Groups* (Healthy, Fatigued MS patients) as between-subjects factor.

The control group consisted of 12 healthy volunteers (9 females, 3 males, mean age 35 ± 8 years and age range 26–49 years), who did not undergo neuromodulation. They were matched for age (independent sample t-test p = 0.654) and gender with the fatigued MS subgroup of 15 people (patients below 50 years old, 11 females, 4 males, age 36 ± 8 years and age range 24–50 years).

The Cortical District*HealthyFatiguedMS Groups effect [F(1,25) = 6.790; p = 0.015] revealed a peculiar difference in the organization of FS_S1_ with respect to FS_M1_ networks among fatigued patients and controls. After applying the Reduced Models for the two cortical regions separately, FS_S1_ FD turned out to be greater in fatigued MS patients than controls and such a difference was more prominent in the left than right hemisphere [F(1,25) = 4.670; p = 0.040]. Conversely, FS_M1_ FDs did not show a difference in MS patients with respect to controls (p = 0.300). Of note, the left FS_S1_ FD, mostly changed by FaReMuS, was differed in patients from controls before FaReMuS treatment (Table 2, p = 0.082) and this difference disappeared after the treatment (p = 0.978). Finally, we note that while in healthy control FS_S1_ FD is smaller than FS_M1_ FD (Table 2, paired sample t-test t(11) = −7.775, p < 0.001), this differentiation was lost in pre-FaReMuS left hemisphere (p = 0.919) and was regained after FaReMuS (t(14) = −5.304, p < 0.001).

### FaReMuS effects on functional connectivity of the primary sensorimotor cortex

We studied whether the intra- and inter-hemispheric functional connectivity between FS_S1_ and FS_M1_ areas changed with the treatment. The former (MutInf between S1left-M1left, and S1right-M1right) was studied by the ANOVA model including *FaReMuS Treatment* (Pre, Post) and *Hemisphere* (Left, Right) as within-subject factors. A clear *FaReMuS Treatment* * *Hemisphere* effect emerged [F(1,17) = 11.469, p = 0.004]. When investigating such dynamics at each hemisphere separately, we found that in the left hemisphere the S1-M1 mutual information decreased [*FaReMuS Treatment* F(1,17) = 5.224, p = 0.035; Table 3], and it increased in the right hemisphere [F(1,17) = 7.265, p = 0.015; Table 3].

**Table 3:** Functional connectivity before and after FaReMuS.

	Mutual Information							
	Real				Sham			
	S1-M1 Left	S1-M1 Right	S1^L^-S1^R^	M1^L^ -M1^R^	S1-M1 Left	S1-M1 Right	S1^L^-S1^R^	M1^L^ -M1^R^
Pre FaReMuS	0.30	0.16	0.12	**0.11**	0.13	0.12	0.14	0.11
	*0.09*	*0.05*	*0.04*	*0.01*	*0.03*	*0.02*	*0.06*	*0.02*
Post FaReMuS	0.26	0.23	0.13	**0.13**	0.11	0.13	0.12	0.11
	*0.08*	*0.12*	*0.03*	*0.02*	*0.02*	*0.04*	*0.04*	*0.02*
Healthy controls	0.25	0.15	0.14	0.13				
	*0.18*	*0.06*	*0.2*	*0.02*				

Mean and *standard deviation* of functional connectivity before and after treatment, for Real and Sham FaReMuS in fatigued MS patients. For formatting and clarity, we omitted FS in the names of the sources. In bold, the values changed after with respect to before FaReMuS for p < 0.010.

To study the functional connectivity between the homologous regions of the two hemispheres, the ANOVA model included *FaReMuS Treatment* (Pre, Post) and *Cortical District* ($F{S}_{S1}$, $F{S}_{M1}$) as within-subject factors. The *FaReMuS Treatment* effect [F(1,17) = 12.402, p = 0.003] indicated that the overall mutual information (MutInf) between left and right FS_S1_ and between left and right M1 increased after FaReMuS. Although the difference in the response between the two cortical regions was not statistically significant [interaction factor *FaReMuS Treatment* * *Cortical District* [F(1,17) = 2.659, p = 0.121], the functional connectivity varied mostly **between left and right FS**_**M1**_ [***FaReMuS Treatment*** effect [F(1,17) = 22.061**, p = 0.0002**] (Table 3). Instead, the functional connectivity between left and right FS_S1_ did not change (*FaReMuS Treatment* effect [F(1,17) = 1.314, p = 0.267], Table 3).

We compared the MutInf between left and right FS_M1_ after Sham and Real stimulations by the ANOVA with *FaReMuS Treatment* (Pre, Post) and *RealSham* (Real, Sham) within-subject factors. A strong difference of the effects of Real and Sham stimulations [FaReMuS Treatment*RealSham interaction effect F(1,11) = 13.235, p = 0.004], corresponded to the above described increase of functional connectivity between left and right FS_M1_ after Real treatment (p = 0.0002), which was absent after Sham (p = 0.274). The pre-treatment functional connectivity values did not differ between Real and Sham blocks [*RealSham* factor F(1,11) = 1.822, p = 0.204].

To better understand the direction of the functional connectivity changes, we compared the functional connectivity between homologous FS_M1_ regions with healthy control values. Fatigued patients had lower pre-treatment values (Table 3 and Fig. 3, independent t-test t(25) = 4.587, p < 0.001), whereas no post-treatment difference was detected between the two groups (Table 3 and Fig. 3, t(25) = 0.412, p = 0.684).

### Relationship of brain factors modified by FaReMuS with its efficacy against fatigue

Those brain features that changed after FaReMuS treatment (p < 0.01, i.e. left FS_S1_ FD and MutInf between left and right M1) were correlated with the mFIS difference (pre-post) divided by the mFIS pre-treatment to describe the percentage change of fatigue levels.

The percentage ameliorations of fatigue levels correlated with the changes of left FS_S1_ fractal dimension (Pearson’s rho = 0.613, p = 0.009 with total mFIS) and with the post-treatment FS_M1_-FS_M1_ functional connectivity (rho = 0.594, p = 0.009). While they correlated with each other (rho = 0.495, p = 0.037), both variables entered the regression model with mFIS percentage change as dependent variable and $F{S}_{S1}^{L}$ FD change and post-FaReMuS FS_M1_-FS_M1_ connectivity as independent variables (FS_M1_-FS_M1_ MutInf):

$$\begin{array}{rcl}{\rm{mFIS}}\,{\rm{percentage}}\,{\rm{change}} & = & -\,22+940.9\,\ast \,{\rm{FD}}(F{S}_{S1}^{L})\\  &  & +469.5\,\ast \,{\rm{MutInf}}\,(F{S}_{M1}^{L},\,F{S}_{M1}^{R}\,)\end{array}$$

In particular, the 48% of mFIS percent change variance was explained by this model (F(2,15) = 6.930, p = 0.007). The coefficients of the model showed that higher values of both variables (i.e. change of $F{S}_{S1}^{L}$ FD and post-FaReMuS FS_M1_-FS_M1_ functional connectivity) predict higher fatigue amelioration.

### Behavioral effects

We submitted the 9 Hope Peg Test execution times to the ANOVA with *Hand* (left, right) and *FaReMuS* (pre, post) within-subject factors. The analysis showed a strong expected dominance effect, with the execution time with the right dominant hand shorter than with the left hand (Table 4; F(1, 14) = 19.365, p = 0.001). Instead, the analysis did not show either the *FaReMuS* effect or the FaReMuS*Hand interaction (both p > 0.200), thus not supporting the shortened time by FaReMuS, and at a stronger level for the dominant hand, suggested by the average values.

**Table 4:** Fine hand movement skill.

		Hand [s]	
		Right	Left
FaReMuS	Pre	19.0 ± 1.2	20.7 ± 1.2
	Post	18.4 ± 1.0	20.3 ± 1.0

9 Hole Peg Test execution time (s) with the right and left hand, before and after FaReMuS treatment.

## Discussion

Double is the most relevant finding of our investigation:

1. FaReMuS reduced the functional imbalance within the primary sensorimotor network, in a way that explains a significant part of its fatigue-relieving potential;
2. before treatment, S1 shows more severely impaired resting state dynamics than M1 in fatigued people with MS.

### FaReMuS effects on sensorimotor network activity and on fatigue

FaReMuS treatment targeting whole body S1 was able to modify the local neurodynamics more at S1 than at M1 cortex. Overall, the left S1 region, which showed the strongest alteration before treatment, turned out to be the region with the highest change after the neuromodulation, and with the capability to restore values to normal physiology. Moreover, S1 neurodynamics recovered the physiological differentiation from M1 as a consequence of the intervention^22^.

FaReMuS neuromodulation intervention modified the functional connectivity among the four main nodes within the primary sensorimotor network bilaterally. This was achieved by re-balancing the inter-hemispheric pre-treatment difference between the left and right S1-M1 interplay resulting in a reinforcement of the functional interplay between homologous M1 areas. As assessed by the regression model, the final M1-M1 connectivity contributed to the fatigue symptoms amelioration, underlining the key role played by the continuity of the tonic interplay between cortical homologs.

Of note, although the same stimulation was delivered to homologous hemispheric areas with an equidistant occipital reference, the effects were unexpectedly asymmetric: the fractal dimension of S1 neurodynamics was diminished in the left dominant hemisphere, and not contralaterally. These findings strongly underline that the effects of neuromodulation depends on the state of the target networks, hence caution is mandatory when extrapolating from healthy controls the expected effects in people suffering from a neuronal damage (MS fatigue here). In fact, when healthy people were stimulated bilaterally by the same electrodes, comparable effects were found in both hemispheres^7^, without differences in the induced local effects between the bilateral^38^ and the monolateral stimulation^39^.

Here, we specifically quantified the functional features - neurodynamics and functional connectivity in resting state - of the bilateral primary somatosensory and motor cortices. We are aware that this selective assessment is a limit of the present investigation, which we plan to overcome in future developing other analysis approaches to assess the activity of the complete cortical mantle.

### S1 more than M1 alteration pre-treatment

In the attempt to alleviate fatigue in people with MS, we adapted a tDCS treatment, which improved the endurance to fatigue when applied in healthy people. In physiological condition, tDCS focused onto both M1 and S1 regions^40^, while in fatigued people with MS we targeted tDCS onto the whole body S1 representation against an occipital electrode^6,7^. This choice was made according to literature data showing that somatosensory^41,42^ and parietal regions^43,44^ display poorer activity and greater atrophy in fatigued MS patients with respect to healthy controls and non-fatigued people with MS. Here we documented a higher resting state pre-treatment impairment of S1 with respect to M1, as compared to control values, providing novel signs of an impoverished local organization of the dominant somatosensory hand representation in MS fatigue. Previously, in MS patients the distortion of the intra-cortical connectivity was specifically observed in the left dominant hemisphere^42^. Overall, these findings support the notion of a specific role in the fatigue-related mechanisms played by the parietal cortex, with the local impairment more evident in the dominant control regions. Such an involvement strengthens the notion that an altered perception due to functional and structural disconnections in the brain parenchyma because of MS plays a crucial part in the generation of fatigue, thus downgrading the competing hypothesis of a decreased motivational drive behind the perception of fatigue^45^. The dominance-driven, hemispheric functional damage may be postulated as a further sign of MS as a disconnection syndrome^46^ that calls into question the more frequently involved networks. The fractal dimension of the local activity is an innovative approach that provides information within a single primary area.

### Fractal dimension to assess resting state activity

In fatigued people with MS, left S1 FD at rest was impaired with respect to healthy controls before the treatment and the difference disappeared after FaReMuS, which modified left S1 FD in a correlated manner with its efficacy against fatigue. Furthermore, the physiological relationship, with a larger FD in M1 than in S1, was lost before FaReMuS, and substantially reverted after the treatment.

Of note, the variability (i.e., irregularity of the spike trains and the spikes counts in invasive recordings and signal fluctuations in non-invasive ones) as well as the irreproducibility of train structures evoked by identical input series, is among the most prominent properties of both spontaneous and evoked neural activity^47^. The fractal dimension, among the complexity measures, is suitable to quantify the recurrence of specific patterns at different scales. The understanding of brain (dys)functionality will benefit of an integrated framework that links brain connectivity to brain dynamics^35^: in this context, the fractal dimension of the local activity can offer a measure of relevant functional properties of the cerebral networking organization at the single node level.

Present data confirms FD as a good candidate for assessing the brain networks functionality^25–28^ and for typifying cortical districts^22,29^.

## Conclusions

This proof-of-principle study showed that the sensorimotor networks activity at rest in fatigued MS people was modulated after the personalized FaReMuS 5-day non-pharmacological treatment in a way that partly explained the fatigue symptoms amelioration. The neuronal activity of the primary sensorimotor counterpart was altered more than the motor one before the treatment. The neuromodulation can effectively revert the unbalanced functional connectivity among interconnected target networks involved in the fatigue symptom.

## Data Availability

EEG raw data, FSS algorithms, personal and clinical anonymized data will be available upon reasonable request.

## References

[1] Kesselring J, Beer S (2005). Symptomatic therapy and neurorehabilitation in multiple sclerosis. Lancet Neurol.

[2] Thompson AJ (1998). Symptomatic treatment in multiple sclerosis. Curr Opin Neurol.

[3] Yusuf A, Koski L (2013). A qualitative review of the neurophysiological underpinnings of fatigue in multiple sclerosis. J. Neurol. Sci..

[4] Tecchio, F. *et al*. Multiple sclerosis fatigue relief by bilateral somatosensory cortex neuromodulation. *J. Neurol*. **261** (2014). 10.1007/s00415-014-7377-9 24854634

[5] Cancelli, A. *et al*. Personalized bilateral whole body somatosensory cortex stimulation to relieve fatigue in multiple sclerosis. *Mult Scler* (2017). 10.1177/1352458517720528 28756744

[6] Tecchio F (2013). Regional personalized electrodes to select transcranial current stimulation target. Front Hum Neurosci.

[7] Cancelli, A., Cottone, C., Di Giorgio, M., Carducci, F. & Tecchio, F. Personalizing the electrode to neuromodulate an extended cortical region. *Brain Stim* (2015). 10.1016/j.brs.2015.01.398 25680321

[8] Parazzini M (2017). A computational model of the electric field distribution due to regional personalized or nonpersonalized electrodes to select transcranial electric stimulation target. IEEE Trans. Biomed. Eng..

[9] Cancelli, A. *et al*. MRI-guided regional personalized electrical stimulation in multisession and home treatments. *Front. Neurosci*. **12** (2018). 10.3389/fnins.2018.00284 PMC5964158 29867308

[10] Ferrucci R (2014). Transcranial direct current stimulation (tDCS) for fatigue in multiple sclerosis. NeuroRehabilitation.

[11] Saiote C (2014). Impact of transcranial direct current stimulation on fatigue in multiple sclerosis. Restor Neurol Neurosci.

[12] Cohen, J. *Statistical Power Analsis of the Behavioral Sciences*. *Lawrence Earlbaum Associates*, 10.1234/12345678 (1988).

[13] Sawilowsky Shlomo S. (2009). New Effect Size Rules of Thumb. Journal of Modern Applied Statistical Methods.

[14] Tecchio F, Porcaro C, Barbati G, Zappasodi F (2007). Functional source separation and hand cortical representation for a brain-computer interface feature extraction. J Physiol.

[15] Porcaro, C. & Tecchio, F. Semi-blind Functional Source Separation Algorithm from Non-invasive Electrophysiology to Neuroimaging. In *Blind**Source**Separation*. (ed. Springer) 521–551 (2014).

[16] Porcaro C, Barbati G, Zappasodi F, Rossini PM, Tecchio F (2008). Hand sensory-motor cortical network assessed by functional source separation. Hum Brain Mapp.

[17] Tecchio F (2008). High-gamma band activity of primary hand cortical areas: a sensorimotor feedback efficiency index. Neuroimage.

[18] Betti V, Zappasodi F, Rossini PM, Aglioti SM, Tecchio F (2009). Synchronous with your feelings: sensorimotor {gamma} band and empathy for pain. J Neurosci.

[19] Melgari JM (2013). Movement-induced uncoupling of primary sensory and motor areas in focal task-specific hand dystonia. Neuroscience.

[20] Porcaro C (2013). Multiple frequency functional connectivity in the hand somatosensory network: an EEG study. Clin Neurophysiol.

[21] Porcaro Camillo, Cottone Carlo, Cancelli Andrea, Salustri Carlo, Tecchio Franca (2018). Functional Semi-Blind Source Separation Identifies Primary Motor Area Without Active Motor Execution. International Journal of Neural Systems.

[22] Cottone Carlo, Porcaro Camillo, Cancelli Andrea, Olejarczyk Elzbieta, Salustri Carlo, Tecchio Franca (2016). Neuronal electrical ongoing activity as a signature of cortical areas. Brain Structure and Function.

[23] Cottone Carlo, Cancelli Andrea, Pasqualetti Patrizio, Porcaro Camillo, Salustri Carlo, Tecchio Franca (2017). A New, High-Efficacy, Noninvasive Transcranial Electric Stimulation Tuned to Local Neurodynamics. The Journal of Neuroscience.

[24] Higuchi T (1988). Approach to an irregular time series on the basis of the fractal theory. Phys. D.

[25] Di Ieva A, Esteban F, Grizzi F, Klonowski W, Martín-Landrove M (2015). Fractals in the neurosciences, Part II: clinical applications and future perspectives. Neuroscientist.

[26] Zappasodi F, Marzetti L, Olejarczyk E, Tecchio F, Pizzella V (2015). Age-Related Changes in Electroencephalographic Signal Complexity. PLoS One.

[27] Zappasodi Filippo, Olejarczyk Elzbieta, Marzetti Laura, Assenza Giovanni, Pizzella Vittorio, Tecchio Franca (2014). Fractal Dimension of EEG Activity Senses Neuronal Impairment in Acute Stroke. PLoS ONE.

[28] Smits FM (2016). Electroencephalographic Fractal Dimension in Healthy Ageing and Alzheimer’s Disease. PLoS One.

[29] Marino Marco, Liu Quanying, Samogin Jessica, Tecchio Franca, Cottone Carlo, Mantini Dante, Porcaro Camillo (2018). Neuronal dynamics enable the functional differentiation of resting state networks in the human brain. Human Brain Mapping.

[30] Pereda E, Quiroga RQ, Bhattacharya J (2005). Nonlinear multivariate analysis of neurophysiological signals. Progress in Neurobiology.

[31] Buyukturkoglu K (2017). Simple index of functional connectivity at rest in Multiple Sclerosis fatigue. Clin Neurophysiol.

[32] Cogliati Dezza I (2015). Functional and structural balances of homologous sensorimotor regions in multiple sclerosis fatigue. J. Neurol..

[33] Torabi Ali, Daliri Mohammad Reza, Sabzposhan Seyyed Hojjat (2017). Diagnosis of multiple sclerosis from EEG signals using nonlinear methods. Australasian Physical & Engineering Sciences in Medicine.

[34] Engel A. (1997). Role of the temporal domain for response selection and perceptual binding. Cerebral Cortex.

[35] Kopell NJ, Gritton HJ, Whittington MA, Kramer MA (2014). Beyond the connectome: The dynome. Neuron.

[36] McDonald WI (2001). Recommended diagnostic criteria for multiple sclerosis: guidelines from the International Panel on the diagnosis of multiple sclerosis. Ann Neurol.

[37] Cohen, M. X. *Analyzing Neural Time Series**Data: Theory and Practice*. *MIT Press*, 10.1017/CBO9781107415324.004 (2014).

[38] Cancelli, A. *et al*. Cortical inhibition and excitation by bilateral transcranial alternating current stimulation. *Restor Neurol Neurosci*, 10.3233/RNN-140411 (2015). 10.3233/RNN-140411 25588458

[39] Moliadze V, Atalay D, Antal A, Paulus W (2012). Close to threshold transcranial electrical stimulation preferentially activates inhibitory networks before switching to excitation with higher intensities. Brain Stimul.

[40] Cogiamanian F, Marceglia S, Ardolino G, Barbieri S, Priori A (2007). Improved isometric force endurance after transcranial direct current stimulation over the human motor cortical areas. Eur J Neurosci.

[41] Dell’Acqua ML (2010). Thalamocortical sensorimotor circuit in multiple sclerosis: an integrated structural and electrophysiological assessment. Hum Brain Mapp.

[42] Tecchio F (2008). Intra-cortical connectivity in multiple sclerosis: a neurophysiological approach. Brain.

[43] Pellicano C (2010). Relationship of cortical atrophy to fatigue in patients with multiple sclerosis. Arch Neurol.

[44] Vecchio F (2017). Electroencephalography-Derived Sensory and Motor Network Topology in Multiple Sclerosis Fatigue. Neurorehabil Neural Repair.

[45] de Lange FP (2004). Neural correlates of the chronic fatigue syndrome–an fMRI study. Brain.

[46] Granberg T (2017). *In vivo* characterization of cortical and white matter neuroaxonal pathology in early multiple sclerosis. Brain.

[47] Moutard C, Dehaene S, Malach R (2015). Spontaneous Fluctuations and Non-linear Ignitions: Two Dynamic Faces of Cortical Recurrent Loops. Neuron.

